# Cerebral metabolic covariance in delirium: pattern response to symptomatic changes

**DOI:** 10.1002/alz.70993

**Published:** 2025-12-15

**Authors:** Sean J. Colloby, Anita Nitchingham, Sarah Richardson, Rachel A. Lawson, John T. O'Brien, Eva A. Wegner, Robert Welschinger, Gideon A. Caplan, John‐Paul Taylor

**Affiliations:** ^1^ Translational and Clinical Research Institute, Faculty of Medical Sciences Newcastle University Newcastle upon Tyne UK; ^2^ Department of Geriatric Medicine Prince of Wales Hospital Sydney Australia; ^3^ Neuroscience Research Australia University of New South Wales Sydney Australia; ^4^ AGE Research Group, Translational and Clinical Research Institute, Faculty of Medical Sciences Newcastle University Newcastle upon Tyne UK; ^5^ Department of Psychiatry University of Cambridge School of Clinical Medicine Cambridge UK; ^6^ Nuclear Medicine Department Prince of Wales Hospital Sydney Australia

**Keywords:** ^18^F‐fluorodeoxyglucose positron emission tomography, cerebral glucose metabolism, delirium, dementia, neuroimaging, spatial covariance

## Abstract

**BACKGROUND:**

Delirium is an acute neuropsychiatric condition linked to increased dementia risk, yet its mechanisms remain unclear. Previous studies reported cerebral hypometabolism. Spatial covariance of ^18^F‐fluorodeoxyglucose (FDG) positron emission tomography (PET) was applied to acutely unwell inpatients with and without delirium (Delirium, Con_unwell_) to derive a delirium‐specific metabolic pattern (DP). DP expression was evaluated in healthy older adults (Con_healthy_) and patients with delirium superimposed on dementia (DSD) and tracked longitudinally with symptom resolution.

**METHODS:**

Seventy participants were included (30 Con_healthy_, 10 Con_unwell,_ 13 Delirium, 17 DSD). Voxel principal components (PCs) identified intercorrelated metabolic patterns.

**RESULTS:**

The DP distinguished Con_unwell_ from Delirium, with relative hypometabolism in default, frontoparietal, visual, and frontostriatal networks, and relative hypermetabolism in sensorimotor and limbic hubs. Delirium and DSD demonstrated higher DP expression than controls. In participants with follow‐up, recovery was paralleled by reduced DP expression.

**DISCUSSION:**

Delirium exhibited a metabolic profile of network dysfunction that may be modifiable and clinically responsive.

**Highlights:**

- Derivation of a DP, primarily attributable to delirium itself, rather than acute illness or dementia.
- The DP revealed metabolic dysfunction spanning large‐scale networks, consistent with the proposed model of delirium as global brain failure.
- DP expression was elevated in delirium, both with and without dementia, compared to healthy and acutely unwell controls.
- In participants with follow‐up, clinical recovery was paralleled by a reduction in DP expression.
- The delirium pattern reflected clinically responsive and modifiable network dysfunction.

## 1 BACKGROUND

Delirium is a serious acute neuropsychiatric condition associated with altered attention and arousal.^1^ It affects up to 40% of hospitalized patients over the age of 65 and is associated with increased risk of mortality.^2^ Despite this, the underlying neurobiological mechanisms of delirium remain unclear. Dementia is a significant risk factor for delirium,^3^ and delirium itself predicts the onset of dementia, with repeated episodes linked to worse cognitive outcomes.^4^, ^5^ Evidence of axonal injury during delirium also supports a potential causal relationship with dementia.^6^ Notably, studies have indicated that prior delirium episodes are more common in individuals who develop dementia with Lewy bodies (DLB) compared to Alzheimer's disease (AD).^7^, ^8^ Fluctuations in attention and alertness, a core clinical feature of DLB, can resemble episodes of delirium, and in some older adults, delirium onset may be the earliest manifestation of prodromal DLB.^9^

Effective management strategies to prevent or treat delirium and potentially modify the risk of incident dementia are lacking.^10^ Investigating the neurobiology of delirium and identifying potential therapeutic targets could not only improve patient outcomes from delirium but also help reduce the incidence of dementia. Even with the apparent shared clinical presentations between delirium and dementia, such as impaired cognition and fluctuating symptoms, pathology studies suggest that pathways associated with cognitive decline in the post‐delirium period differ from those in dementia,^11^ so it is important to be able to characterize those commonalities and divergences.

Decreased cortical glucose metabolism is a hallmark of dementia, where tracers such as ^18^F‐fluorodeoxyglucose (FDG), a glucose analog, serve as a surrogate measure of glucose metabolism and a reliable indicator of neural and synaptic activity. ^18^F‐FDG positron emission tomography (PET) imaging can be used to diagnose and assess progression in a number of dementia subtypes, including AD, DLB, and frontotemporal dementia (FTD).^12^ Decreased cerebral metabolism and perfusion have been shown to be associated with delirium^13^, ^14^, ^15^, ^16^; however, some of these investigations were limited by not being able to control for potential confounders, including dementia and illness severity, heterogeneous delirium etiology, or inclusion of only post‐operative delirium participants.^14^, ^15^

Analysis of ^18^F‐FDG PET scans to identify regional changes between selected groups has typically implemented mass‐univariate approaches, which assume each region of interest (ROI) or voxel across the brain to be independent of each other. However, since brain network activity plays an important role in the symptomatology of neurodegenerative disorders,^17^ it is important to consider alterations in brain function at the system network level. One way to examine this is by multivariate approaches such as spatial covariance, a form of principal component analysis (PCA), which overcomes the concept of functional segregation and provides connectivity information between brain regions. Such procedures have been successfully applied to ^18^F‐FDG PET studies in dementia (AD, DLB)^18^, ^19^, ^20^ and in Parkinson's disease.^21^, ^22^, ^23^, ^24^

Identification of a delirium‐specific metabolic pattern may advance mechanistic understanding and clinical management by revealing neural network dysfunctions underlying transient symptomatic disturbances.^25^ It could also clarify how systemic insults disrupt brain metabolism,^26^ enable biological subtyping (e.g., inflammatory or neurotransmitter‐driven forms),^27^ improve prognosis, and provide a reproducible marker for assessing therapeutic response and disease progression. In this study, we undertook a spatial covariance analysis of ^18^F‐FDG PET scans in acutely unwell patients with and without delirium (Delirium, Con_unwell_), none of whom had evidence of dementia. Our aim was to derive a metabolic spatial covariance pattern that differentiated these groups, that is, a delirium‐related pattern (DP), a pattern reflecting metabolic alterations specifically attributable to delirium rather than to acute illness or underlying dementia. To contextualize findings, we then examined the expression of this DP in two independent cohorts: healthy older individuals without acute illness (Con_healthy_) and patients with delirium superimposed on dementia (DSD), comparing them with the Delirium and Con_unwell_ groups. Further, in a minority of participants with repeat PET scanning, we assessed changes in DP expressions at baseline and 3 months in response to the symptomatic changes over this period associated with delirium resolution.

## 2 METHODS

### 2.1 Participants

Participants from two cohorts were included as part of a UK–Australia collaborative study. The UK cohort involved 30 Conhealthy individuals, obtained from a previous ^18^F‐FDG PET dementia study,^28^ all without symptoms of dementia or evidence of acute illness. The study was approved by the Newcastle and North Tyneside Research Ethics Committee (REF 09/H0906/88), and all gave written informed consent. The Australian cohort comprised 10 Con_unwell_, 13 Delirium, and 17 DSD patients. With regard to the types of acute illness in the Con_unwell_ group, nine had sepsis and one had a pelvic fracture.

The Delirium and DSD groups included participants with ongoing delirium formally diagnosed by an experienced geriatrician using the Confusion Assessment Method (CAM).^29^ The presence or absence of dementia at baseline was ascertained using the Informant Questionnaire on Cognitive Decline in the Elderly (IQ‐CODE),^30^ with a cut point of >3.44 indicating dementia.^31^ The criteria for Con_unwell_ were age ≥ 65 years, rated as negative on the CAM, no history of delirium or dementia, Mini‐Mental State Examination (MMSE) score ≥ 26, and acutely unwell and hospitalized. Patients were excluded if they had a history of stroke, traumatic brain injury, or other organic brain pathology that would likely affect the FDG PET scan.^16^ Patients suffering from major depression, patients using regular antipsychotics and anticonvulsants, and patients with poor glycemic control were also excluded due to the potential of these conditions to alter cerebral metabolism on FDG‐PET.^16^ We also omitted cases of delirium tremens and hepatic encephalopathy. Written informed consent for study participation was obtained from all participants with capacity. For participants without capacity, proxy consent was sought from the person responsible according to the New South Wales Guardianship Act (1987).

### 2.2 Assessments

On enrollment in the study, baseline demographic data, including age and sex, were recorded in both cohorts. Participants also completed the MMSE as a measure of global cognition.^32^

In the Australian cohort, an experienced geriatrician confirmed the presence or absence of delirium on the day of the scan using the CAM. Delirium severity was assessed using the Delirium Index.^33^ Acute Physiology and Chronic Health Evaluation (APACHE III)^34^ quantified acute illness severity, while the Charlson Comorbidity Index^35^ quantified the burden of chronic disease. Lastly, the Barthel and Instrumental Activities of Daily Living (iADL) indices^36^, ^37^ evaluated the functional status of participants.

### 2.3 PET acquisitions

All participants underwent ^18^F‐FDG PET scanning, whose acquisition details have been described elsewhere.^16^, ^28^ In brief, for Con_unwell_, Delirium, and DSD groups, patients received an intravenous dose of 3.5 MBq.kg^−1^ of ^18^F‐FDG, followed by imaging for 10 min in a Philips Ingenuity TF128 PET/CT scanner. Philips software used iterative reconstruction with corrections for decay, random coincidences, and scatter to generate the PET scans. For Con_healthy_, participants received an intravenous dose of 250 MBq of ^18^F‐FDG, followed by imaging for 10 min in a Siemens Biograph Truepoint PET/computed tomography (CT) scanner. Siemens software used iterative reconstruction with scatter and attenuation correction to produce the PET data.

**RESEARCH IN CONTEXT**

1. **Systematic review**: The authors reviewed the literature from traditional (e.g., PubMed) sources and online content. Delirium and dementia are closely linked: Dementia increases delirium risk, while delirium predicts dementia onset, and repeated episodes worsen cognition. Associations with reduced cerebral metabolism and perfusion have been reported, though many studies are limited by confounders such as coexisting dementia, illness severity, heterogeneity of etiology, or restriction to post‐operative cohorts. Defining delirium's neurobiology and therapeutic targets may improve outcomes and reduce the incidence of dementia.
2. **Interpretation**: We identified a DP of glucose metabolism, applicable to independent groups and repeated imaging. This supports the use of DP expression as a marker to monitor brain changes during symptomatic progression and recovery.
3. **Future directions**: Validation of the DP in larger cohorts is required, along with defining network hubs most sensitive to delirium and testing its ability to distinguish dementia from DSD.

### 2.4 Spatial preprocessing

All PET scans were spatially normalized to match an aging‐dementia‐specific ^18^F‐FDG PET template in standard stereotactic space using SPM12 (https://www.fil.ion.ucl.ac.uk/spm/). The aging‐dementia PET template was derived from 100 images (50 controls, 50 patients), details of which are reported.^38^ Spatial normalization utilized a 12‐parameter affine transformation followed by non‐linear warping (7 × 9 × 7 basis functions with 16 iterations). The SPM12 parameter defaults for image registration were as follows: no template and source weighting, 25 mm cutoff, medium regularization, preserve concentration, trilinear interpolation, and bounding box equal to template image. For participants with repeat ^18^F‐FDG PET scanning, a rigid‐body (six‐parameter) realignment between their repeat and unregistered baseline scans was performed, followed by application of the transformation parameters, estimated from the spatial normalization of the baseline scan and aging‐dementia PET template, to the realigned repeat scan. Registration accuracy was visually checked at each stage. All spatially transformed images had dimensions of 79 × 95 × 78, with an isotropic voxel size of 2 mm, upon which each scan was then smoothed with a 10‐mm FWHM 3D Gaussian filter.

### 2.5 Spatial covariance

Spatial covariance analysis was performed on “*n*” preprocessed (registered and smoothed) ^18^F‐FDG PET scans using covariance software (http://www.nitrc.org/projects/gcva_pca/).^39^ This approach identified the main sources of variation, yielding (*n* − 1) principal component (PC) images ranked in descending order of explained variance. A mask image defined the brain volume subspace for voxel analyses. To control for individual differences in global tracer uptake, each participant's global mean (within the brain mask) was computed and subtracted from the data matrix prior to PCA. For each PC image, voxels had positive and negative weights representing the sign and strength of voxel covariance that remained fixed across participants. Specifically, positive and negative voxels were interpreted as concomitant relative increased and decreased glucose metabolism, respectively. The degree to which a participant expressed a PC image (PC_1_, PC_2_,…, PC*_n_*_−1_) was by means of the subject‐scaling factor (SSF_1_, SSF_2_,…, SSF*_n_*_−1_), formed from effectively the “dot product” (PC_i_ ∙ PET_FDG_) of two images, yielding a scalar value (SSF_i_) representing the projection of each PC_i_ onto an individual's FDG‐PET scan. Therefore, a high SSF_i_ score indicates a greater normalized increased metabolism in voxels with positive weights and a greater normalized decreased metabolism in voxels with negative weights.

To derive the metabolic spatial covariance pattern (SCP_FDG_) that discriminated Con_unwell_ from Delirium patients, all SSF scores (SSF_1_, SSF_2_,…, SSF*_n_*_−1_) were entered into a linear regression model as independent variables with “group” as the dependent measure. Akaike's information criterion (AIC) determined how many SSFs (PCs) should be included in the regression model to achieve an optimum trade‐off between goodness of fit and model simplicity.^40^ The set of SSFs (PCs) generating the lowest AIC value were chosen as predictors for the model, where the resulting linear combination formed the composite SCP_FDG_ or delirium pattern (DP). The extent to which each participant expressed the DP was by the SSF_DP_, that is, SSF_DP_ = DP ∙ PET_FDG_ of two scans, representing the projection of the DP onto each individual's FDG‐PET image.

The stability and reliability of the DP were assessed by bootstrap resampling (1000 iterations) to identify areas that contributed to the pattern with high confidence. This transforms the voxel weights of the DP into Z maps, computed as the ratio of voxel weight and bootstrap standard deviation. The Z‐statistic follows roughly a standard normal distribution where a one‐tailed *p* ≤ 0.05 suggests a threshold of |Z| ≥ 1.64.^41^ Anatomical labeling of the Z maps used the MRIcroGL image visualization software (https://www.nitrc.org/projects/mricrogl), along with the “aal3” brain atlas.^42^

### 2.6 Statistical analyses

Analysis used IBM SPSS version 29.0.1.0 and R (version 4.0.3, https://www.R‐project.org/). Variables were tested for normality and variance homogeneity using Shapiro–Wilk and Levene's tests, respectively. The data were examined using parametric (ANOVA F, Welch's ANOVA W) and non‐parametric (Mann–Whitney U, χ^2^) tests. Correlations were assessed with Pearson coefficients (*r*). Where appropriate, Benjamini–Hochberg multiple‐comparisons correction (*p*’) was applied with a false discovery of *Q* < 0.05.

## 3 RESULTS

### 3.1 Participant demographics and clinical characteristics

Table 1 shows demographic and clinical characteristics. Inpatient groups (Delirium, DSD, and Con_unwell_) were similar with respect to sex, age, APACHE III, Charlson, and Barthel indices. Global cognition scores (MMSE) were significantly higher in both control groups compared to the delirium participants with and without dementia (*p* < 0.001), while DSD participants had poorer global cognition compared to those with delirium without dementia (*p* = 0.04). DSD participants had greater functional iADL dependency compared to Con_unwell_ participants, while IQ‐CODE values were greater for DSD compared to delirium and Con_unwell_ groups (*p* < 0.001).

**TABLE 1:** Baseline demographic and clinical data for groups studied with 18F‐FDG PET.

	Con_healthy_	Con_unwell_	Delirium	DSD	Statistic, *p* value
*N*	30	10	13	17	
Sex (m:f)	20:10	7:3	5:8	7:10	χ^2^_(3) _= 5.3, *p* = 0.2
Age	76.3 ± 6.6	84.5 ± 7.3	84.5 ± 4.5	86.7 ± 5.5	**F_3,66_ = 13.1, *p* < 0.001** ^a^
MMSE	28.9 ± 1.1	28.3 ± 1.1	18.9 ± 5.6	13.5 ± 5.9	**W_3,24.2_ = 48.5, *p* < 0.001** ^b^
Delirium type (hypo:hyper:mixed)	–	NA	2:1:10	4:2:11	χ^2^_(2) _= 0.5, *p* = 0.8
Delirium Index	–	NA	8.6 ± 3.5	9.8 ± 3.3	F_1,28_ = 0.9, *p* = 0.3
APACHE III score	–	34.8 ± 13.3	37.5 ± 10.5	38.9 ± 6.1	F_2,37_ = 0.6, *p* = 0.6
Duration of delirium at scan (days)	–	NA	11.9 ± 16.1	9.6 ± 6.8	*U* = 128, *Z* = 0.7, *p* = 0.5
IQ‐CODE score	–	3.2 ± 0.2	3.4 ± 0.2	4.1 ± 0.6	**W_2,24.3_ = 16.8, *p* < 0.001** ^c^
Charlson Comorbidity Index	–	5.4 ± 2.0	6.1 ± 2.7	6.4 ± 1.9	F_2,36_ = 0.7, *p* = 0.5
Barthel Index	–	19.1 ± 2.2	16.7 ± 5.6	16.5 ± 4.4	H_2_ = 3.4, *p* = 0.2
iADL Index	–	10.6 ± 3.7	7.2 ± 3.5	4.4 ± 3.5	**F_2,35_ = 9.1, *p* < 0.001** ^d^

*Note*: Data expressed as mean ± 1 SD. Bold text denotes statistical significance. –, not measured.

Abbreviations: APACHE III, Acute Physiology and Chronic Health Evaluation; DSD, delirium superimposed on dementia; FDG, fluorodeoxyglucose; iADL, Instrumental Activities of Daily Living; IQ‐CODE, Informant Questionnaire on Cognitive Decline in the Elderly; MMSE, Mini‐Mental State Examination; PET, positron emission tomography; SD, standard deviation.

Post‐Hoc tests.

^a^ Gabriel's: Con_healthy_ < Con_unwell_, Delirium, DSD (*p* ≤ 0.002).

^b^ Games–Howell: Con_healthy_, Con_unwell_ > Delirium, DSD (*p* < 0.001); Delirium > DSD (*p* = 0.07).

^c^ Games–Howell: DSD > Con_unwell_, Delirium (*p* < 0.001).

^d^ Gabriel's: Con_unwell_ > DSD (*p* < 0.001).

### 3.2 Spatial covariance

**FIGURE 1:**
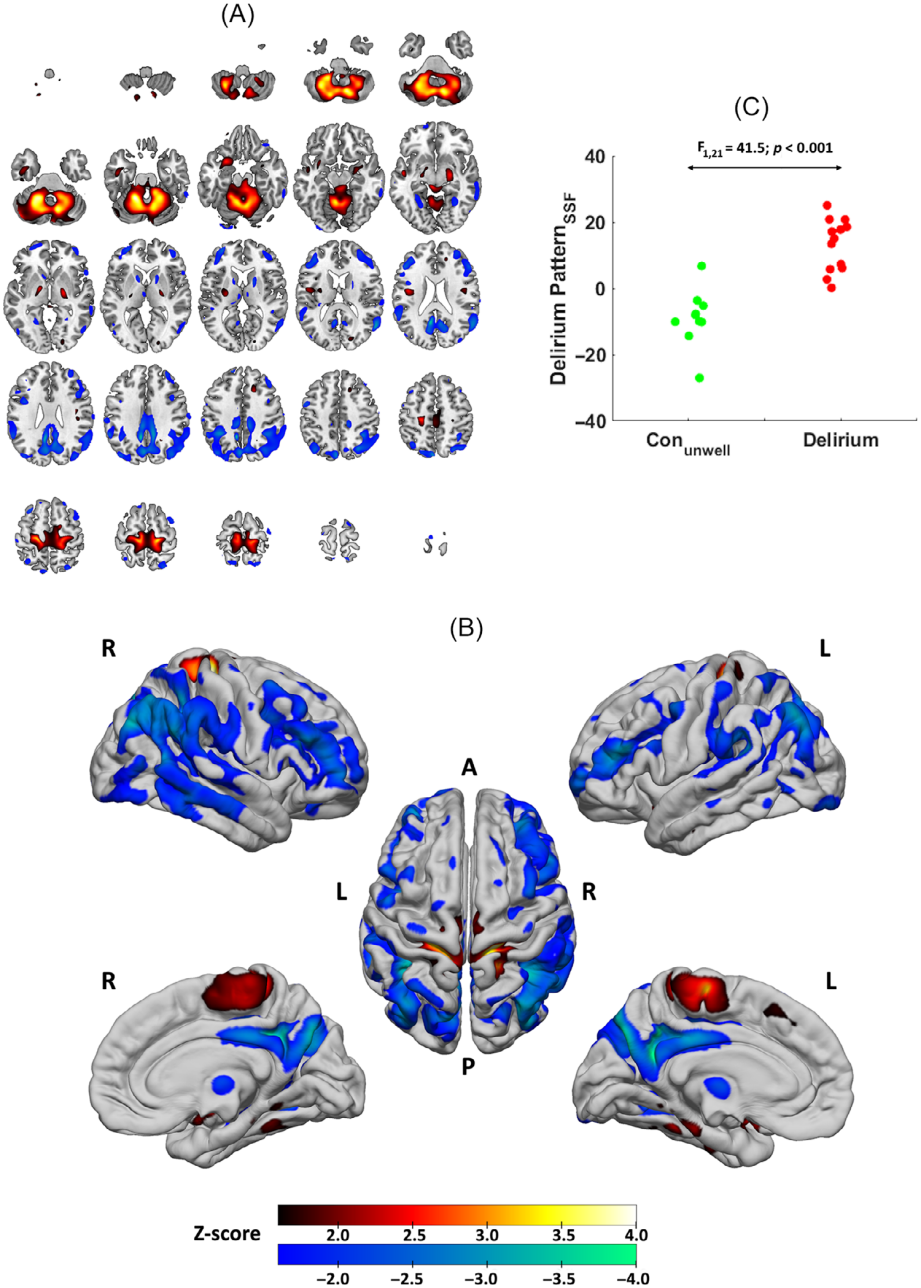
^18^F‐FDG PET metabolic pattern associated with delirium projected onto axial (A) and cortical surface rendered magnetic resonance imaging brain template (B), with scatter plot of participant scores (DP_SSF_) versus group (C). Images are displayed neurologically (Left is left, A = anterior, P = posterior). DP_SSF_, subject‐scaling factor of delirium‐specific metabolic pattern; FDG, fluorodeoxyglucose; PET, positron emission tomography.

The DP, a composite image of PC_1_, PC_2_, PC_5_, and PC_6_, differentiated Delirium from Con_unwell_ (Figure 1a,b). SSF_DP_ scores, representing the extent to which participants expressed the topography, were higher in the delirium without dementia group than in Con_unwell_ (mean ± SD: Delirium = 13.2 ± 7.9, Con_unwell_ = −8.9 ± 8.5; F_1,21_ = 41.5, *p* < 0.001; Figure 1c). The pattern was characterized by concomitant decreased metabolism (blue) in inferior/middle/superior temporal gyrus, fusiform, lingual/cuneus/precuneus, middle occipital gyrus, the inferior/middle/superior frontal gyrus, caudate, mediodorsal thalamus, mid/posterior cingulate, and inferior/superior parietal lobe, with concomitant increased metabolism (red) in the cerebellum, supplementary motor areas, precentral, paracentral, amygdala, and parahippocampal structures. The decreased uptake pattern implicated regions within default mode, frontoparietal, visual, language, and frontostriatal networks, while the increased pattern involved regions within sensorimotor and limbic hubs. Table 2 depicts details of specific regions contributing to the DP with high confidence (|Z| ≥ 1.64, *p* ≤ 0.05).

**TABLE 2 alz70993-tbl-0002:** Regions contributing to DP with high confidence (|Z| ≥ 1.64, *p* ≤ 0.05).

Hemisphere	MNI coordinates	Region	*Z*‐score
R	65, −40, −19	Inferior temporal gyrus	−1.7
R	65, −38, −13	Middle temporal gyrus	−1.8
L	−57, −40, 20	Superior temporal gyrus	−2.0
R	59, −51, 19	Superior temporal gyrus	−2.2
L	−29, −48, −10	Fusiform	−1.8
R	28, −54, −10	Fusiform	−1.9
L	−26, −52, −7	Lingual gyrus	−1.8
R	26, −50, −7	Lingual gyrus	−1.7
L	−38, −82, 31	Middle occipital gyrus	−2.2
R	43, −73, 31	Middle occipital gyrus	−1.8
L	−8, −65, 22	Cuneus	−2.4
R	11, −68, 22	Cuneus	−2.3
L	−7, −57, 19	Precuneus	−3.1
R	5, −59, 19	Precuneus	−2.4
L	−42, 39, 13	Inferior frontal gyrus	−2.0
R	49, 31, 19	Inferior frontal gyrus	−1.9
L	−37, 53, 8	Middle frontal gyrus	−2.2
R	42, 54, 0	Middle frontal gyrus	−2.1
L	−18, 64, −6	Superior frontal gyrus	−1.9
R	34, 51, 13	Superior frontal gyrus	−2.0
L	−12, 14, 0	Caudate	−1.9
R	13, 13, 0	Caudate	−1.9
L	−5, −16, 8	Mediodorsal magnocellular thalamus	−1.8
R	6, −13, 8	Mediodorsal magnocellular thalamus	−1.7
L	−6, −51, 28	Posterior cingulate	−3.2
R	8, −47, 28	Posterior cingulate	−2.2
L	−6, −33, 36	Midcingulate	−2.7
R	8, −34, 36	Midcingulate	−2.5
L	−40, −69, 44	Angular gyrus	−2.1
R	53, −60, 31	Angular gyrus	−1.9
L	−57, −48, 28	Supramarginal gyrus	−2.3
R	62, −48, 28	Supramarginal gyrus	−2.1
L	−40, −44, 38	Inferior parietal	−2.3
R	42, −44, 38	Inferior parietal	−2.4
L	−14, −77, 51	Superior parietal	−2.0
R	35, −64, 51	Superior parietal	−2.0
L	−27, −55, −43	Cerebellum	3.5
R	29, −57, −43	Cerebellum	3.0
L	−7, −19, 60	SMA	2.1
R	6, −19, 60	SMA	1.8
L	−20, −27, 67	Precentral gyrus	2.1
R	16, −32, 67	Precentral gyrus	2.8
L	−10, −27, 67	Paracentral lobule	3.2
R	16, −30, 67	Paracentral lobule	3.1
L	−18, −28, −20	Parahippocampal gyrus	1.8
R	30, 2, −16	Amygdala	1.8

### 3.3 Forward projections of DP

**FIGURE 2 alz70993-fig-0002:**
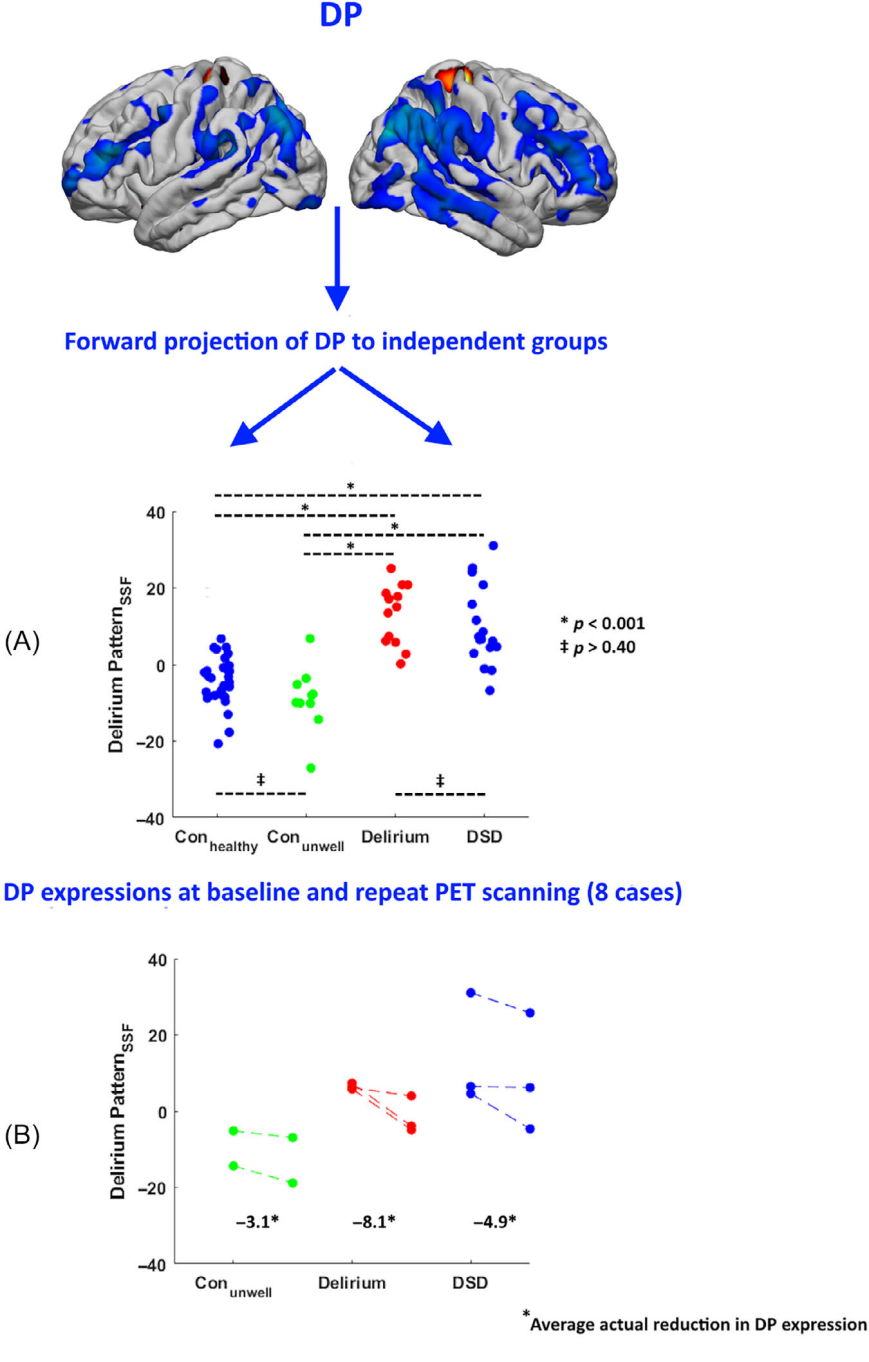
Scatter plots depicting pattern expressions from forward projections of metabolic DP applied to independent groups (Con_healthy_, DSD) (A) and a minority of cases with repeat scanning (two Con_unwell_, three Delirium, three DSD) (B). Con_unwell_, acutely unwell without delirium; DP, delirium‐specific metabolic pattern; DSD, delirium superimposed on dementia; SMA, supplementary motor area.

We then examined to what extent two independent groups, Con_healthy_ and DSD participants, expressed the DP relative to acutely unwell patients with and without delirium without dementia (Delirium, Con_unwell_). This was performed by computation of the SSF_DP_ scores, that is, DP ∙ PET_FDG_, in Con_healthy_ and DSD, where Figure 2a shows their distributions relative to Delirium and Con_unwell_. Table 3 details the average SSF_DP_ scores across groups, with Delirium and DSD both demonstrating significantly higher DP expressions compared to Con_healthy_ and Con_unwell_ (*p* < 0.001). Further, DP expression did not differ between delirium with and without dementia (Delirium vs DSD), or between acutely unwell patients and healthy controls (*p* ≥ 0.4).

**TABLE 3 alz70993-tbl-0003:** DP expressions across groups.

	Con_healthy_	Con_unwell_	Delirium	DSD	Statistic, *p*‐value
*N*	30	10	13	17	
DP expression	−3.5 ± 7.5	−8.9 ± 8.5	13.2 ± 7.9	9.8 ± 10.4	**F_3,66_ = 21.9, *p* < 0.001** ^a^

*Note*: Data expressed as mean ± 1 SD. Bold text denotes statistical significance. Results based on Sidak post hoc tests.

Abbreviations: DSD, delirium superimposed on dementia

^a^ Con_healthy_, Con_unwell_ < Delirium, DSD (*p* < 0.001).

Con_healthy_ versus Con_unwell_ (*p* = 0.4).

Delirium versus DSD (*p* = 0.9).

DP expression values were also computed for the eight participants who underwent repeat PET imaging at 3‐month follow‐up. Figure 2b illustrates DP expression scores at baseline and repeat in eight participants (two Con_unwell_, three Delirium, three DSD). Table 4 summarizes the symptom and cognitive data of each participant with follow‐up at each time point, demonstrating that all participants showed clinical improvement in terms of acute illness, global cognition, function, and, for participants with delirium, delirium severity. In all cases, symptomatic improvement was accompanied by a reduction in DP expression, where the average ΔSSF_DP_ scores were −3.1, −8.1, and −4.9 in Con_unwell_, Delirium, and DSD, respectively.

**TABLE 4 alz70993-tbl-0004:** Changes in clinical characteristics in individuals with baseline and 3‐month repeat imaging.

	Con_unwell__1	Con_unwell__2	Del_1	Del_2	Del_3	DSD_1	DSD_2	DSD_3
MMSE_baseline_	29	28	26	18	21	5	15	16
MMSE_3months_	29	30	29	26	28	13	26	20
% ΔMMSE^b^	0	7.1	11.5	44.4	33.3	160.0	73.3	25.0
Del index_baseline_	0	0	7	10	7	13	10	9
Del index_3months_	0	0	0	2	2	4	1	6
% ΔDel index^b^			−100.0	−80.0	−71.4	−69.2	−90.0	−33.3
APACHE III_baseline_	22	31	25	23	35	41	44	31
APACHE III_3months_	17	17	–	–	35	27	24	20
% ΔAPACHE III^b^	−22.7	−45.2			0	−34.1	−45.5	−35.5
Barthel_baseline_	18	20	20	18	19	12	20	12
Barthel_3months_	18	20	–	18	20	13	14	16
% ΔBarthel^b^	0	0		0	5.3	8.3	−30.0	33.3
iADL_baseline_	12	12	9	5	9	0	10	0
iADL_3months_	12	12	–	5	7	0	2	0
% ΔiADL^b^	0	0		0	−22.2		−80.0	
SSF_DP_baseline_	−5.2	−14.3	5.8	6.2	7.4	31.1	6.5	4.6
SSF_DP_3months_	−6.9	−18.8	−4.9	4.0	−3.9	25.8	6.2	−4.6
ΔSSF_DP_ ^a^	−1.7	−4.5	−10.7	−2.2	−11.3	−5.3	−0.3	−9.2
% ΔSSF_DP_ ^b^	−32.7	−31.5	−184.5	−35.5	−152.7	−17.0	−4.6	−200.0

*Note*: Missing data (–).

Abbreviations: APACHE III, Acute Physiology and Chronic Health Evaluation; Del, Delirium; DSD, delirium superimposed on dementia; iADL, Instrumental Activities of Daily Living; MMSE, Mini‐Mental State Examination; SSF, subject‐scaling factor.

^a^ (3months – baseline) score.

^b^ (3months – baseline)/|baseline| × 100%.

## 4 DISCUSSION

For the first time, this study identified a spatial covariance pattern of metabolic dysfunction associated with delirium, implicating disruption in large‐scale networks such as default mode, frontoparietal, visual, language, and frontostriatal, consistent with the concept of delirium as global brain failure. DP expression was elevated in delirium, both with and without dementia, compared to healthy and acutely unwell controls, while no significant differences were observed between the two delirium groups (with and without dementia) or between the two control groups (acutely unwell and well). This supports its specificity to delirium rather than acute illness or underlying dementia. Further, in a subset of participants with longitudinal assessment, reduction in DP expression was observed alongside improvements in acute illness, delirium severity, global cognition, and function.

The observed changes in brain metabolism appear to be associated with clinical recovery and may, while only shown in a small number of cases and therefore speculative, serve as a potential marker for identifying delirium and monitoring its progression and resolution, as well as exploring novel treatment strategies.

The derived DP discriminated unwell patients with and without delirium. The DP consisted of concomitant decreased and increased metabolism in several brain regions, where the decreased uptake pattern implicated regions within default mode, frontoparietal, visual, language, and frontostriatal circuits, while the increased pattern involved regions within sensorimotor and limbic hubs. This pattern demonstrated significant disruption in global brain function, which supports previous hypotheses that delirium represents a global brain failure.^43^ These established brain networks support specific processes that correspond to the core domains in delirium, that is, disturbances in attention/awareness and cognitive and psychomotor impairment.^1^, ^43^, ^44^ Default and frontoparietal hubs have roles in attentional control and goal‐directed attention, respectively. Consistent with a functional magnetic resonance imaging (fMRI) study that reported disruptions in reciprocity between default (posterior cingulate cortex) and frontoparietal (dorsolateral prefrontal cortex) nodes in inpatients with delirium, this suggests their involvement in attention and awareness in older adults with delirium.^45^ Others have shown disintegrated, less efficient resting‐state fMRI networks during an episode of delirium, including loss of default hub function (right posterior cingulate cortex).^46^ Cognitive impairment, such as memory and executive functions, have also been associated with frontostriatal, default, and visual network hub dysfunction from fMRI and electroencephalogram assessments in delirium.^47^, ^48^ Hypermetabolism observed in sensorimotor and limbic circuits may underlie hyperactive symptoms such as agitation, restlessness, and anxiety; however, this was outside the scope of this study, and interpretation requires confirmation in studies focusing on patients with hyperactive versus hypoactive delirium at the time of PET scanning. The DP provided novel network‐level understanding that extended previous region‐based analyses of these participants.^16^ The findings highlight the complex nature of delirium and suggest that examining the functional alterations can help identify brain systems that are particularly vulnerable to delirium, as well as those with potential for recovery. Overall, effective management strategies for delirium remain limited, particularly in patients with DSD, and the present results may help with the identification of novel therapeutic targets for future interventions.[Table alz70993-tbl-0002], [Fig alz70993-fig-0002], [Table alz70993-tbl-0003], [Table alz70993-tbl-0004]

Forward projection of the DP to healthy controls and DSD participants revealed similar levels of DP expressions between delirium groups but were significantly higher than unwell and healthy control groups. There was no significant difference between the two control groups. This suggests that the DP is specific to delirium, whether superimposed on dementia or not, rather than acute illness. Previous research demonstrated that phenomenologically, there is little difference in the presence or severity of delirium symptoms in those with and without known dementia,^49^, ^50^ which supports our findings and suggests a shared pathophysiology.^51^ However, due to some phenotypic overlap in presentation, DSD can be challenging to diagnose and may be missed, particularly in those with underlying Lewy body disease.^52^, ^53^ Therefore, the DP might have the potential to stratify individuals with delirium in the presence of dementia or not from those with acute illness for research studies, in particular clinical trials.

Forward projection of the DP to eight cases with longitudinal assessment (baseline, 3 months), including PET scanning, was performed. In all cases, symptomatic recovery in acute illness, global cognition, function, and, where observed, delirium severity coincided with reduced DP expression. The average decrease in DP expression from baseline to repeat was largest for Delirium, followed by DSD then Con_unwell_. This indicated that changes representative of reduced metabolic dysfunction, characterized by decreased DP expression, appeared to reflect some degree of clinical restoration, not only in acutely unwell patients with and without delirium but also patients with DSD, and thus could serve as a possible marker for tracking symptomatic progression to recovery. Also, the modifiable nature of the DP may also open the door for the investigation of novel treatment strategies, for example, in clinical trials. Nonetheless, as these interpretations derive from a small number of individuals, they should be viewed at this stage as exploratory.

Although this is the first study to assess functional spatial covariance patterns of brain metabolism in these groups, there were limitations. Healthy participants (Con_healthy_) were, on average, nearly a decade younger than the other groups (Con_unwell_, Delirium, DSD), though the latter cohorts were similarly aged. All scans prior to covariance analysis and pattern expression evaluation underwent both intensity scaling (log transformation) and subsequent intensity normalization relative to their corresponding mean global brain uptake. This approach sought to harmonize count variations arising from differences between scanners and individuals. However, the primary findings and conclusions of this study, namely, the derivation of the DP, its expression in DSD, and longitudinal changes in DP expression in response to symptom recovery, were all based on imaging data from similarly aged groups acquired from the same PET scanner. The healthy participants only acted as a comparator group to contextualize the findings. Across the combined groups (Con_unwell_, Delirium) and (Con_unwell_, Delirium, DSD), age was not associated with DP expression (|r| = 0.03, *p* ≥ 0.84). In the derivation cohort (Con_unwell_, Delirium), age was included as a dependent variable in the spatial covariance analysis to identify a covariance pattern related to age. Although age corresponded to PC_3_, this association was not significant (r = 0.31, *p* = 0.16) and did not contribute to the DP, which comprised PCs 1, 2, 5, and 6. Therefore, age was unlikely to have influenced the derived DP. There was a bias toward hypoactive and mixed types of delirium. Though two patients in the DSD group were hyperactive and received IM sedation immediately prior to scanning, guidelines have indicated that sedation administered typically 15 min prior to scanning does not appear to affect uptake.^54^ None of the patients in the delirium‐only group received this. Carers were also present to reassure patients as needed and helped mitigate the challenges introduced by scanning. Patients with mixed delirium were usually hyperactive overnight and hypoactive at the time of imaging. We acknowledge that the delirium cohorts were relatively modest, reflecting the practical challenges of recruiting acutely unwell patients for neuroimaging studies. This precluded comparing delirium subtypes in this study, which we recommend as a focus for future work. However, the groups were broadly comparable in size to previously reported neuroimaging studies of delirium. The spatial covariance procedure revealed a robust and biologically plausible delirium‐related pattern (DP), consistent with established regions implicated in attention, arousal, and network integration. Importantly, we demonstrated the reproducibility and external validity of the DP by evaluating its expression across independent groups (Con_healthy_ and DSD), supporting its possible generalizability beyond the derivation sample. As participants were acutely unwell, it was not possible to scan potential participants before their admission to hospital; thus, we cannot confirm whether we have identified brain systems that are particularly vulnerable to delirium or whether delirium directly disrupts these brain systems, or both. Finally, we recognize the complexity of performing PET imaging in acutely unwell individuals and those with delirium and/or dementia. Alternative, easier‐to‐implement approaches, such as functional near‐infrared spectroscopy (fNIR), which can indirectly assess spatial brain metabolic change, may be more practical and scalable for clinical trials.^55^

We characterized a delirium‐specific pattern of glucose metabolism that was able to be applied to independent groups, as well as participants with repeated imaging. In the same individuals (Con_unwell_, Delirium), we previously identified regional hypometabolism associated with delirium using a univariate ROI method.^16^ In contrast, the present assessment advanced those findings by utilizing voxel‐wise spatial covariance, which captures distributed, network‐level metabolic patterns rather than isolated regional effects that can often lack spatial sensitivity and specificity. The multivariate approach allowed us to identify a delirium‐related pattern that reflects coordinated metabolic changes across multiple brain regions, providing a system‐level perspective on delirium pathophysiology. Unlike ROI, spatial covariance revealed network dysfunction that may underlie the fluctuating and global nature of delirium. Further, by quantifying individual expressions of the DP, we demonstrated, although tentatively, its potential as a subject‐level marker for delirium severity, progression, and resolution, yielding new insights into the functional topography underlying this syndrome beyond our previous ROI study. Given the responsive nature of the DP, this methodology, following appropriate validation, could also facilitate the exploration of novel treatment strategies. Future research should aim to validate the DP and its utility for monitoring progression of delirium symptoms, as well as determining whether the DP can distinguish between non‐acutely unwell dementia and DSD.

## CONFLICT OF INTEREST STATEMENT

Drs. Colloby, Nitchingham, Richardson, Wegner, and Welschinger report no disclosures. Professor Taylor has been a consultant for Eisai and Kyowa‐Kirin and received honoraria for talks from GE Healthcare and Bial Pharmaceuticals. Professor Caplan has received speaker fees from Pfizer and AstraZeneca, as well as research funding from Kinoxis. Dr. Lawson has received grant funding from Parkinson's UK and the Lewy Body Society and travel expenses from Michael J. Fox Foundation. Unrelated to this work, Professor O'Brien has acted as a consultant for TauRx, Novo Nordisk, Biogen, Roche, Lilly, GE Healthcare, and Okwin and received grants or academic in‐kind support from Avid/Lilly, Merck, UCB, and Alliance Medical. Author disclosures are available in the Supporting Information.

## CONSENT STATEMENT

Written informed consent was obtained from all participants with capacity. For participants without capacity, proxy consent was sought from the person responsible according to the New South Wales Guardianship Act (1987).

## Supporting information



Supporting information
